# Pre-hospital activation of emergency transfusion in trauma

**DOI:** 10.1186/1757-7241-22-S1-P9

**Published:** 2014-07-07

**Authors:** Tom Evens, Jimmy Siu, Malcolm Russell, Magnus Nelson

**Affiliations:** 1 Kent, Surrey & Sussex Air Ambulance, Redhill, UK

## Background

Haemostatic resuscitation is an important component of the Novel Hybrid Resuscitation strategy. This approach aims to maintain tissue oxygenation, and to counter traumatic coagulopathy maintaining the circulating with blood products which in combination resemble whole blood.

Major Trauma Centres (MTCs) have adopted protocolised rapid access to appropriate blood products, triggered by recognition of ongoing significant bleeding. In some regions this protocol can be requested by pre-hospital services, some of which have the capacity to begin transfusion in the field.

In our Helicopter Emergency Medical Service (HEMS) region, we access five MTCs. All allow HEMS teams to activate their protocol from scene. Each has a different protocol and pack specification.

## Method

We surveyed all MTCs in England to establish current practice and inform further debate regarding transfusion in the pre-hospital setting.

## Results

26/28 MTCs responded. 100% (26/26) reported a written emergency transfusion protocol. 46% (12/26) utilised the triple activation criteria of systolic blood pressure below 90mmHg, active haemorrhage, and failure to respond to a fluid bolus described by Khan et al [1]. A further 38% (10/26) utilised one or two of these criteria. The remainder activated on the decision of the team leader. 12 centres allowed pre-hospital physicians to remotely activate the protocol, and two of these also allowed critical care paramedics to do so.

There was wide variation in the contents of pack A. 3 centres supplied only packed red cells (PRC). 14 centres supplied PRC and fresh frozen plasma (FFP) in a 1:1 ratio. The widest ratio was 8:2. Cyroprecipitate and platelet supply varied.

## Conclusion

There is wide variation in activation and provision of emergency transfusion. The provision of pre-hospital blood, may require MTC’s to adapt the ratio of supplied products.
